# Ramollissement of the Brain

**Published:** 1855-12

**Authors:** 


Lecture III. *Ramollissement of the Brain. and its Differential Diagnosis from Cerebral Hemorrhage.* By Prof. Trosseau.

The present medical generation believes that remollissement and cerebral hemorrhage are two affections characterized by such well-marked symptoms that it is impossible to confound the one with the other. It is established scholastically that, when an individual, whether predisposed or not, suddenly presents the following morbid phenomena, viz: vertigo, loss of consciousness, and paralysis of one of the sides of the body, he is the subject of hemorrhage, on the side of the nervous center opposite to that on which the paralysis has manifested itself. On the other hand, when vertigo, formication, and cramps have existed for more or less time, and paralysis has been gradually established, the lesion is regarded as a ramollissement. As a general rule this is true, but so frequent are the exceptions, that we are unable to found our recognition of the nature of the lesion either upon the mode of invasion or the order of the succession of the symptoms. Thus, while we may sometimes observe paralysis, gradually established after precursory symptoms, arises from sanguineous apoplexy, in other cases a sudden attack of paralysis may be the consequence of ramollissement, as the case in No. 42 testifies.

This woman, aged 42, went to bed, early in February, quite well, and on waking she found that the left side, and especially the arm, were not so strong as the same parts on the right side. Alarmed, she dressed herself with difficulty, and repaired to a doctor, who bled her. While the blood still flowed the patient lost her consciousness, and when she came to, her left side was found paralyzed, and she had to be carried home. The catamenia, which were some days behind their time, re-appeared on the day of the accident. The symptoms of paralysis gradually diminished, and at the next menstrual epoch only a little numbness remained. On the day the menses re appeared the woman was again suddenly seized with paralysis of the left side, and she was then brought to the Hospital.

The following was her condition on admission. She was of a feeble lymphatico-nervous constitution, having the muscular system little developed, and but little *embonpoint.* The left arm hung by her side, the fore-arm was semi-flexed, and the hand in a state of forced pronation, the thumb and fingers being forcibly flexed upon the palm. She could perform no movement with her arm. The leg was less paralyzed than the arm. She could still flex the thigh on the pelvis, and the leg on the thigh, but she could not raise her heel from the bed. All the left side of the face was paralyzed and puffed out, the labial commissure being drawn to the right side, but no deviation occurring to the tongue. The bladder was paralyzed, the catheter being required until death; and the stools came away involuntarily. Sensibility remained unimpaired, and neither vision nor hearing was impaired. Lastly, the understanding was unclouded, the patient relating her history with the greatest clearness.

For some days after her admission the symptoms of paralysis seemed to diminish. She became enabled to execute some movements of the arm, and the urine passed from the catheter with a jet. So she continued until the catamenia re-appeared, when her intellects became enfeebled, and all voluntary motion of the arm or leg was impossible. In the night of the 19th of April these parts were seized with clonic convulsions; the face became red and injected, the pulse full and frequent, and the skin hot and sweating. On the morning of the 20th she was found in the deepest coma, with stertorous breathing. All the right side, the motions of which had, till then, been preserved, was in a complete state of resolution. She died in the night.

*Autopsy thirty-six hours after Death.*—The membranes of the brain were highly injected, and especially on the right side; and the pia mater was firmly adherent to the brain. In one of the anfractuosities of the middle lobe, in the vicinity of the fissure of Sylvius, a notable quantity of pus was found. The convolutions of the anterior lobe, on the same side, were softened especially at the base, and the cerebral substance was carried away by a stream of water. In the central parts two points of white ramollissement were found, the one in the optic thalamus and the other in the corpus striatum. No where could any hemorrhagic effusion be detected, not even those little sanguineous sugillations which are regarded as indicative of capillary hemorrhage. The color of the cerebrum had undergone no change at the level of the softened points, and the parts exhibited in no wise the yellowish tint attributed to hemorrhagiparous ramollissement.

What ought to be our diagnosis in such a case as this? Would not the following be the classical one? Hemorrhage had taken place in the cerebral substance of the right side, which was renewed at the recurrence of the menstrual epoch. Lastly, in accordance with the ideas established by Lallemand, around this hemorrhagic deposit the cerebral substance became inflamed, thus explaining the re-actional phenomena which terminated the scene. This was the diagnosis indicated, and the one which, in the great generality of cases, would have proved true. A strong motive for believing in the existence of sanguineous apoplexy was that these accidents occurred every time in the midst of the hemorrhagic molimen of menstruation. I found myself inclined towards the common opinion, although I felt that there were powerful reasons for suspending my judgment—reasons that I derived from the nature of the paralytic accidents themselves. With a most complete hemiplegia as regards movement, the general sensibility, the senses, and the intellect were preserved unimpaired. Now, I remembered to have heard Recamier teach, and I have since observed it to be so myself, that when there is dissonance among the symptoms they are due to ramollissement, while, on the other hand, we must conclude that a hemorrhage exists when there is a consonance in the paralytic phenomena. That which in our case seemed a reason for suspending the judgment, namely, the hemorrhagic molimen that preceded the paralysis, only adds to the force of the distinction laid down by Recamier; and it remains demonstrated, at any rate, that a sudden paralysis of a portion of the body may be the result of a ramollissement.—*London Lancet.*

